# Symptom-to-balloon time and myocardial blush grade are predictors of left ventricular remodelling after successful primary percutaneous coronary intervention

**DOI:** 10.5830/CVJA-2016-085

**Published:** 2017

**Authors:** El-Sayed M Farag, Mohammad M Al-Daydamony

**Affiliations:** Cardiology Department, Faculty of Medicine, Zagazig University, Zagazig, Al-Sharkia, Egypt; Cardiology Department, Faculty of Medicine, Zagazig University, Zagazig, Al-Sharkia, Egypt

**Keywords:** primary PCI, left ventricular remodelling, myocardial blush, symptom-to-balloon time

## Abstract

**Introduction:**

In patients with ST-segment elevation myocardial infarction (STEMI), successful primary percutaneous coronary intervention (PCI) was found to be useful in earlier restoration of TIMI flow 3. However, the incidence of left ventricular (LV) dilatation and remodelling after successful primary PCI is still high. We aimed to determine the independent predictors of LV remodelling after successful primary PCI for patients with first STEMI.

**Methods:**

We included 232 STEMI patients treated with primary PCI. Echocardiography was done on the day of PCI and after six months. LV remodelling was defined as ≥ 20% increase in the six-month left ventricular end-diastolic volume (LVEDV).

**Results:**

In patients with remodelling, symptom-to-door and symptom-to-balloon times were significantly longer (p < 0.00001 for each), initial ejection fraction (EF) was significantly lower (p = 0.044), six-month LVEDV, left ventricular end-systolic volume (LVESV) and LVEDV increase were significantly higher, and EF was significantly lower (p < 0.00001 for each). Mean myocardial blush grade (MBG) was significantly lower in patients with remodelling (p < 0.00001). There was a significant positive correlation between LVEDV increase and both symptom-to-balloon time (r = 0.603, p < 0.00001) and symptom-to-door time (r = 0.564, p < 0.00001), and a significant negative correlation between LVEDV increase and MBG (r = –0.447, p < 0.00001). Logistic regression showed that the independent predictors of LV remodelling were symptom-to-balloon time (p = 0.00068), symptom to door time (p = 0.0013) and MBG (p = 0.0057).

**Conclusion:**

Symptom-to-door time, symptom-to-balloon time and MBG were the only significant predictors of LV remodelling.

## Introduction

ST-segment elevation myocardial infarction (STEMI) is one of the most important causes of death and disability around the world.^1^ Heart failure (HF) is a serious sequel of STEMI. Left ventricular (LV) remodelling was found to be the precursor to developing HF and also an important predictor of prognosis after STEMI.^2^

When compared with fibrinolytic therapy for STEMI patients, successful primary percutaneous coronary intervention (PCI) was found to be useful in earlier restoration of thrombolysis in myocardial infarction (TIMI) flow grade 3 flow in the infarctrelated artery, it limited the infarction size, and decreased heart failure and mortality rates.^3^ However, the incidence of LV dilatation after successful primary PCI is still high.^4^

Previous studies have searched for predictors of LV remodelling after primary PCI. Regional and global LV systolic dysfunction, severe LV diastolic abnormalities,^5^ lower LV ejection fraction at discharge,^6^ and poorer myocardial perfusion as assessed by myocardial blush grade (MBG)^6^, ^7^ were found to be significant predictors of LV remodelling. However, these studies were performed on relatively small numbers of patients. The aim of our study was to determine the independent predictors of LV remodelling after successful primary PCI for patients with first STEMI.

## Methods

This prospective study was done in the coronary care and cardiac catheterisation units of the Cardiology Department, Zagazig University. The study population consisted of 260 patients who were admitted with acute STEMI during the period between January 2012 and January 2015.

The inclusion criteria were: confirmed acute STEMI, defined as the presence of typical chest pain that lasts for at least 20 minutes, and ST-segment elevation ≥ 0.1 mV in at least two contiguous leads;^8^ primary PCI done within 12 hours of the onset of symptoms; successfully performed PCI with < 20% residual stenosis and TIMI flow 3 of the infarct-related artery defined as normal flow, which fills the distal coronary bed completely.^9^

Patients were excluded from our study in the presence of one or more of the following: previous history of coronary artery disease (CAD), myocardial infarction, or revascularisation; more than mild valvular stenosis or regurgitation; patients with left bundle branch block; and unsatisfactory echocardiographic images.

We had a written informed consent from every patient. The study protocol was approved by the institutional review board of the Faculty of Medicine, Zagazig University 

A full history was taken and a complete clinical examination was done on every patient. The time of onset of chest pain (symptom time), the time of the patient’s arrival at the hospital (door time), and the time of first balloon inflation or stent deployment (balloon time) were carefully recorded.

Symptom-to-door time was defined as the interval between the appearance of symptoms and arrival at the hospital. Doorto- balloon time was defined as the interval between the arrival at hospital and the time of balloon inflation. Symptom-to-balloon time was defined as the interval between the onset of symptoms and the time of balloon inflation.^10^

Complete standard 12-lead electrocardiography was carried out on each patient. Echocardiographic studies were performed on all patients using the GE VIVID E9 machine with 2.5-MHz transducers. The studies were performed by two operators unaware of each other’s measures and of the patients’ clinical and angiographic data. Views were taken while the patients were in the left lateral position.

Left ventricular end-diastolic volume (LVEDV) and end-systolic volume (LVESV) were measured from the apical two-chamber and apical four-chamber views. Ejection fraction (EF) was calculated using the Simpson’s method.11 Echocardiography was done within 24 hours of admission, and was repeated after six months. LV remodelling was defined as ≥ 20% increase in the six-month LVEDV.^5^

Primary PCI was performed on all patients within 12 hours of onset of symptoms by two expert interventionists; at least one met the criteria of individual operator level volume of the 2007 Clinical Competence Statement on Cardiac Interventional Procedures and its 2013 revision.^12^ Stenting, balloon dilatation and/or thrombus aspiration were done, and glycoprotein (GP) IIb/IIIa inhibitor (eptifibatide) was given as appropriate, according to operator opinion.

TIMI flow and myocardial blush grade (MBG) were assessed by two expert angiographers unaware of each other’s results and of the patients’ other data. MBG was assessed according to the dye density score as follows: MBG 0 = no myocardial blush or contrast density, MBG 1 = minimal myocardial blush or contrast density, MBG 2 = moderate myocardial blush or contrast density but less than that obtained during angiography of a contralateral or ipsilateral non-infarctrelated coronary artery, MBG 3 = normal myocardial blush or contrast density, comparable with that obtained during angiography of a contralateral or ipsilateral non-infarctrelated coronary artery.^13^

After discharge, all patients continued to receive medical treatment, including aspirin, clopidogrel, beta-blockers, statins, aldosterone antagonists and angiotensin converting enzyme inhibitors (ACEIs) or angiotensin receptors blockers (ARBs).^8^

## Statistical analysis

All data were analysed using the SPSS for windows package (Version 20.0; Armonk, NY, USA: IBM Corp). Differences between the study groups were analysed using the χ2 and student’s t-tests. Correlations between different variables were investigated by Pearson correlation analysis. The logistic regression analysis was evaluated by the Hosmer–Lemes goodness-of-fit test. A p-value < 0.05 was regarded as being statistically significant.

In order to assess the intra-observer variability, we repeated the measures offline for echocardiography and angiography in 30 patients within seven days of the first measure. The interand intra-observer variability were calculated by dividing the difference between the two sets of measurements by the mean of the two observations.

## Results

Twenty-eight more patients were excluded from the initial study group (three patients died, five had a non-fatal MI and were excluded to avoid the effect of a second infarction on LV remodelling, five patients underwent revascularisation before the second echocardiogram, and 15 patients were not adherent with follow up). The remaining 232 patients (121 males and 111 females) constituted the study group.

LV remodelling was detected in 68 patients (29.3%). Patients were divided into two groups according to the presence or absence of LV remodelling.

Regarding clinical and echocardiographic data, and as shown in Table 1, there was no significant difference between the two groups regarding age, gender, diabetes, hypertension, smoking, dyslipidaemia, door-to-balloon time, incidence of anterior infarction, peak CK-MB level, troponin T level, basal LVEDV or basal LVESV.

**Table 1:** Clinical and echocardiographic data

*Parameters*	*Remodelling (n = 68)*	*No remodelling (n = 164)*	*p-value*
Age (years)	58.4 ± 9.73	56.5 ± 10.85	0.193
Gender, n (%)			
Male	37 (54.4)	84 (51.2)	0.659
Female	31 (45.6)	80 (48.8)	
Diabetes, n (%)	22 (32.3)	49 (29.9)	0.71
Hypertension, n (%)	28 (41.2)	60 (36.6)	0.512
Smoking, n (%)	20 (29.4)	53 (31.5)	0.664
Dyslipidaemia, n (%)	23 (33.8)	55 (33.5)	0.966
Symptom-to-door time (min)	380.2 ± 105.1	289.5 ± 85.6	< 0.00001
Door-to-balloon time (min)	44.5 ± 10.6	46.8 ± 11.2	0.14
Symptom-to-balloon time (min)	424.1 ± 107.3	335.8 ± 93.1	< 0.00001
Anterior infarction, n (%)	45 (66.2)	100 (61)	0.267
Peak CK-MB (IU/l)	289.5 ± 102.3	271.3 ± 98.4	0.214
Troponin T (ng/ml)	10.78 ± 3.95	9.85 ± 4.22	0.111
Day 1 LVEDV (ml)	101.3 ± 22.5	95.6 ± 18.8	0.067
Day 1 LVESV (ml)	42 ± 13.6	38.2 ± 14.5	0.059
Day 1 EF (%)	58.4 ± 5.63	60.1 ± 6.22	0.044
6-month LVEDV (ml)	135.6 ± 26.4	103.5 ± 20.1	< 0.00001
6-month LVESV (ml)	65.6 ± 18.5	40.1 ± 16.3	< 0.00001
6-month EF (%)	51.6 ± 9.63	61.2 ± 7.14	< 0.00001
LVEDV increase (%)	33.9 ± 7.53	8.26 ± 6.53	< 0.00001

Data are expressed as mean ± SD or number (%). LVEDV = left ventricular end-diastolic volume, LVESV = left ventricular end-systolic volume, EF = ejection fraction.

Mean EF was significantly lower in patents with remodelling (p = 0.044). After six months, mean LVEDV, LVESV and percentage of LVEDV increase were significantly higher, and mean EF was significantly lower in patients with remodelling (p < 0.00001 for each). Mean symptom-to-door and symptomto- balloon times were significantly higher in patients with remodelling (p < 0.00001 for each).

Regarding primary PCI data, and as shown in Table 2, there was no significant difference between the two groups regarding stenting, thrombus aspiration, use of GP IIb/IIIa inhibitors, infarct-related artery, incidence of patients with multi-vessel disease, mean baseline stenosis, stent diameter, stent length, or final residual stenosis. There was a significant difference between the two groups regarding MBG, with more patients with MBG 0 and 1, and fewer patients with MBG 2 and 3 among patients with remodelling (p < 0.00001). Mean MBG was significantly lower in patients with remodelling (p < 0.00001).

**Table 2:** PCI data

*Parameters*	*Remodelling (n = 68)*	*No remodelling (n = 164)*	*p-value*
Stenting, n (%)	65 (95.6)	161 (98.2)	0.259
Thrombus aspiration, n (%)	23 (33.8)	58 (34.5)	0.823
GPIIb/IIIa inhibitors, n (%)	31 (45.6)	77 (46.9)	0.849
Infarct-related artery, n (%)
LAD	41 (60.3)	93 (56.7)	0.877
LCX	13 (19.1)	35 (21.3)	
RCA	14 (20.6)	36 (22)	
Multi-vessel disease, n (%)	22 (32.3)	50 (29.8)	0.799
Baseline stenosis (%)	95.3 ± 4.61	94.7 ± 5.34	0.391
Stent diameter (mm)	3.12 ± 0.561	3.24 ± 0.644	0.157
Stent length (mm)	15.8 ± 5.74	16.3 ± 6.33	0.559
Final stenosis (%)	5.76 ± 4.22	6.33 ± 5.11	0.381
MBG: 0	28	21	< 0.00001
MBG: 1	17	24	
MBG: 2	11	52	
MBG: 3	12	67	
Mean MBG	1.102 ± 0.913	2.001 ± 1.036	< 0.00001

Data are expressed as mean ± SD or number (%). LAD = left anterior descending artery, LCX = left circumflex artery, RCA = right coronary artery, MBG = myocardial blush grade.

**Fig. 1.**
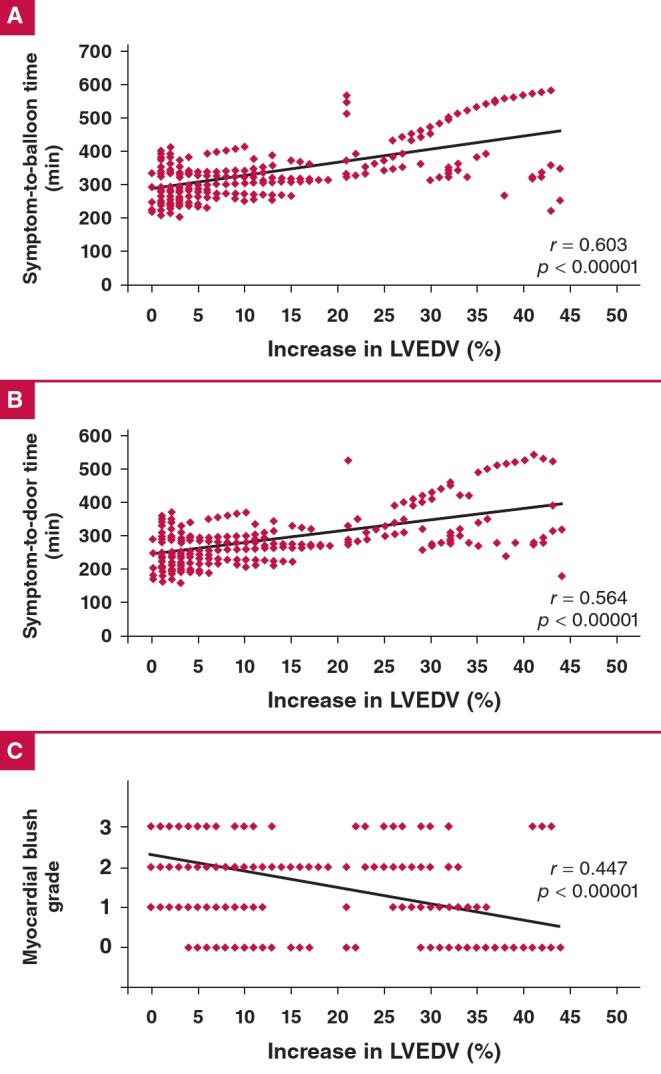
Correlations between increase in LVEDV and symptom-to-balloon time (A), symptom-to-door time (B), and MBG (C).

Fig. 1 shows the correlations between increase in LVEDV and different PCI parameters. There was a significant positive correlation between LVEDV increase and both symptom-toballoon time (r = 0.603, p < 0.00001, Fig. 1A) and symptomto- door time (r = 0.564, p < 0.00001, Fig. 1B). There was a significant negative correlation between LVEDV increase and MGB score (r = –0.447, p < 0.00001, Fig. 1C).

Logistic regression for the independent predictors of LV remodelling is shown in Table 3. The independent predictors of LV remodelling, in order of significance, were symptom-toballoon time (p = 0.00068), symptom-to-door time (p = 0.0013) and MBG (p = 0.0057).

**Table 3:** Logistic regression analysis for independent predictors of LV remodelling

*Variables*	*Odds ratio*	*95% CI*	*p-value*
Symptom-to-balloon time	3.78	2.41–5.15	0.00068
Symptom-to-door time	4.61	3.01–6.21	0.0013
Myocardial blush grade	3.44	1.65–5.22	0.0057
Baseline EF	2.99	1.12–4.86	0.0744
Peak CK-MB	2.71	1.14–4.28	0.1564
Baseline LVEDV	1.3	0.63–1.97	0.253
Age	1.06	0.47–1.65	0.365

CI = confidence interval, LVEDV = left ventricular end-diastolic volume, EF = ejection fraction.

Inter- and intra-observer variability for different echocardiographic parameters ranged from 1.5 to 7.1%. For LVEDV, inter- and intra-observer variability were 5.2 ± 2.7 and 6.2 ± 3.4%, respectively. For MBG score, inter- and intraobserver variability were 2.3 ± 1.1 and 4.5 ± 2.4%, respectively.

## Discussion

In our study, logistic regression analysis showed that the only significant independent predictors for the occurrence of LV remodelling were symptom-to-balloon time, symptom-to-door time and MBG.

The great advantage of primary PCI over thrombolysis is the earlier and the more effective restoration of coronary flow. This in turn leads to fewer incidences of recurrent ischaemia, better LV function, and of course better clinical outcome of STEMI patients treated with primary PCI, compared to those treated with thrombolysis.^3^.

However, even after successful restoration of blood flow in the infarct-related artery after STEMI, a considerable number of patients still suffer from LV dilatation and impairment in systolic function. Among 284 STEMI patients treated with primary PCI, Bolognese and colleagues recorded a 30% incidence of LV remodelling, defined as > 20% LVEDV increase.^4^ This incidence was similar to the 29.3% incidence of LV remodelling in our study group.

Primary PCI significantly improves blood flow in epicardial coronary arteries in STEMI patients. However, this improvement does not always include microvascular flow and myocardial perfusion. After restoration of blood flow in the occluded epicardial artery, some pathophysiological changes tend to occur and may contribute to the impairment in microcirculatory flow. These changes include infiltration of neutrophils, endothelial dysfunction, tissue oedema and microembolisation.^14^

The relationship between microvascular dysfunction and LV function and outcome after primary PCI has been studied by many investigators. Poli and colleagues found that MBG score and ST-segment elevation recovery after successful primary PCI were associated with the degree of early and late recovery of LV function.^15^

The ability of MBG score to predict survival rate was studied by Stone et al.^16^ They found a strong relationship between survival rate and MBG score after primary or rescue PCI. Among their study group, one-year survival rate was 6.8% in patients with normal MBG scores, 13.2% in patients with reduced MBG, and 18.3% in patients with absent blush.

Bolognese et al. found that microvascular dysfunction, as assessed by intracoronary myocardial contrast echo score index, was able to predict the occurrence of LV remodelling as well as unfavourable long-term outcome.^17^ De Luca and colleagues found that MBG score was an independent predictor for one-year mortality after primary PCI for STEMI patients presenting with signs of heart failure.^18^

The value of time to primary PCI was studied by Soon and colleagues. They found that symptom-to-balloon not door-toballoon time was a significant independent predictor of shortand medium-term mortality rates and major adverse cardiac events.^10^ Symptom-to-balloon time was found by Hahn et al. to be associated with infarct transmurality.^19^

Why do some STEMI patients, even after successfully performed primary PCI, have poor myocardial perfusion with MBG 0 or 1? In an attempt to answer this question, Prasad et al. studied the effect of prolonged ischaemia on MBG score after primary PCI. Their main finding was that delayed primary PCI was associated with greater injury to the microcirculation and impaired myocardial perfusion. They also found that patients presenting four hours after symptom onset had a higher incidence of MBG 0 and 1, compared to those presenting within two hours.^20^

Several mechanisms may lead to impaired myocardial perfusion in patients with prolonged ischaemic time. These include endothelial dysfunction and damage, interstitial and cellular haemorrhage/oedema, formation of micro-thrombi within the micro-vessels, and an increase in thrombus organisation with time, which lessens its responsiveness to antiplatelet and anticoagulant therapies and increases the probability for distal macro- or micro-embolisation.^21^,^22^

Earlier researchers found that many factors may influence the process of LV remodelling, such as patency of the infarctrelated artery,^23^ treatment with angiotensin converting enzyme (ACE) inhibitors and/or beta-blockers,^24^ and baseline BNP concentrations.^25^ Treatment with renin–angiotensin–aldosterone blockers after MI was found to ameliorate the process of LV remodelling in experimental models^26^ as well as in humans.^3^,^27^

In our study, which was a single-centre study, all our patients were treated in a similar way. This may explain why symptom-toballoon time, symptom-to-door time and MBG score were the only significant predictors for LV remodelling. Mean door-toballoon time was 46.1 ± 11.8 minutes in the whole study group and it did not differ significantly between the two groups. It also did not correlate with increase in LVEDV. The door-to-needle time was also fairly similar in the whole study group, probably since the study was conducted in a single centre with the same treatment strategy applied to all patients.

The longer symptom-to-door time in the remodelling group made the symptom-to-balloon time significantly longer as well. This may have been caused by many factors, including lack of public awareness of the symptoms of STEMI and the importance of seeking medical help timeously, the small number of centres capable of performing primary PCI, the large distance between these centres and primary health centres, and the huge traffic problem in a developing country such as Egypt.

Limitations of this study are that it was carried out in a single centre, and recording the time of onset of symptoms is totally subjective, which may make measuring of symptom-to-door time inaccurate.

## Conclusion

Our study showed that after successfully performed primary PCI for STEMI patients, symptom-to-door time, symptom-toballoon time and MBG were the only significant predictors of LV remodelling. Efforts must be made to reduce symptom-todoor time, including promoting health awareness of cardiac symptoms, educating primary healthcare providers, and improving the ambulance system.
